# The Association Between Eosinophils and the Disease Process in Destructive Non-invasive Fungal Rhinosinusitis

**DOI:** 10.7759/cureus.46558

**Published:** 2023-10-05

**Authors:** Amal A Almaghrabi, Khaled I Alnoury, Ahmed K Alkhulaifi

**Affiliations:** 1 Otolaryngology, Security Forces Hospital Program, Makkah, SAU; 2 Otolaryngology, King Abdulaziz University, Jeddah, SAU; 3 Faculty of Medicine, King Abdulaziz University, Jeddah, SAU

**Keywords:** prognosis, destructive fungal nasal polyposis, chronic rhinosinusitis, blood levels, eosinophils

## Abstract

Objective: This study aims to investigate the significance of eosinophils in the progression and prognosis of destructive non-invasive fungal rhinosinusitis.

Methods: This was a retrospective study of 126 patients who were operated on for a nasal sinus disease between 2010 and 2017; of these, 56 met the inclusion criteria*. *These were divided into two groups: (i) Group 1, with only the destructive non-invasive sinus polyposis type, and (ii) Group 2, with all types of nasal polyposis other than the destructive non-invasive sinus fungal polyposis type. Data including demographic data, paranasal sinuses (PNS) CT scans, pre- and post-operative eosinophils count, histopathology of polyps, and fungal cultures were collected from medical records from King Abdulaziz University Hospital, Jeddah, Saudi Arabia. A nonparametric Wilcoxon signed-rank test was used to determine a significant difference of p< 0.05. We performed multivariate analysis using repeated measure analysis of covariance (ANCOVA) and adjusted for confounders such as age, sex, pre- and post-operative IgE, fungal culture, and histopathology of the disease. Interaction between age, sex, IgE, and proportion of eosinophils was assessed at a p-value of 0.05.

Results: A significant difference was found between pre-and post-operative blood eosinophils levels in Group 1 (p= 0.01) after adjusting for confounders such as age, sex, fungal culture, pre and post IgE, and histopathology of the disease. However, no significant difference was found in Group 2 (p= 0.663) even after adjusting for age, sex, fungal culture, pre and post IgE, and histopathology of the disease. We did not find any significant interaction (P>0.05) between pre- and post-operative levels of eosinophils with age, sex, and fungal culture among Group 1 and Group 2.

Conclusions: There is a statistically significant difference in blood eosinophils between pre- and post-operative levels in studied cases of destructive non-invasive fungal polyposis, a difference is not seen in nasal sinus polyp cases other than destructive non-invasive fungal polyposis types. High pre-operative eosinophils levels in destructive non-invasive fungal sinus polyposis types demonstrated the importance of eosinophils in the pathogenesis of this disease. The blood eosinophils can therefore be considered an important factor in the disease process and an indicator of the disease prognosis and destructive behavior.

## Introduction

Rhinosinusitis is a common disease affecting approximately 20% of the population [1]. Chronic rhinosinusitis (CRS) is a chronic disease that accounts for >90% of all rhinosinusitis cases [2], and CRS with nasal polyps has an incidence of 20-30% among CRS patients [3], with controversies surrounding the role of fungi in this condition. CRS patients with nasal polyposis are commonly associated with having higher blood eosinophils count than CRS patients without nasal polyposis [4-6], and therefore have different pathological inflammatory pathways, cytokine profiles, and tissue remodeling, resulting in a different clinical presentation [7-9]. Rowe-Jones and Moore Gillon proposed a chronic destructive but non-invasive (semi-invasive) type of rhinosinusitis [10]. The criteria in this type are sinus expansion with bony erosion without histological evidence of tissue invasion [10-12]. 

There is an important relation between eosinophil count and the pathogenesis of the destructive non-invasive fungal disease, as eosinophils play a major role in the onset and progression of destructive behavior fungal sinusitis [13-16]. Various studies demonstrate high levels of toxic Major Basic Protein (MBP) from eosinophils in the mucus of patients with CRS [15-18].

Allergies may be associated with eosinophilic mucin, but eosinophilic mucin can be present independent of patients' atopic or allergic status [19]. Nasal polyposis characterized by eosinophil-dominated inflammation in fungal rhinosinusitis patients is a complex process, and understanding of the disease pathophysiology is limited [4,13,20]. Ponikau et al. showed that fungal growth was found in 96% of patients with CRS with more than 25% of them having type 1 hypersensitivity to fungi [21]. Several studies found that in CRS patients, the activated eosinophils will promote the inflammatory effects of the disease [7,16,22].

To date, there is a lack of published data regarding the causes of the destructive non-invasive nature of fungal nasal polyps. Therefore, this study aims to investigate the significance of eosinophil presence as a major role in the pathogenesis and prognosis of destructive fungal non-invasive nasal polyposis, exploring the possible causes that make the polyp of destructing nature to the tissues without invasion in some patients with fungal nasal polyposis which could help in the management of such diseases.

## Materials and methods

Data collection and study procedures

In this retrospective study, a medical record review was done for 126 nasal sinus disease patients who underwent nasal surgeries between 2010-2017 in King Abdulaziz University Hospital, Jeddah, Saudi Arabia. Male and female patients diagnosed with nasal sinus polyposis diseases and between nine and 65 years of age were included in the study. Fifty-six cases were found to meet these inclusion criteria, which were divided into two groups. The inclusion criteria of the first group (Group 1) were the cases with only destructive non-invasive fungal sinus polyposis type as shown by radiographic studies and histopathology results and evidence of fungal sinus disease. The second group (Group 2) included cases of inflammatory and allergic sinus polyposis, non-invasive fungal polyposis, invasive fungal sinus polyposis, and granulomatous fungal sinus polyposis. Male and female patients diagnosed with non-polyposis nasal sinus diseases and/or given steroids either pre- or post-operatively were excluded from the study.

Demographic data, para nasal sinus (PNS) CT scans, preoperative and one-month post-operative blood eosinophils count percentage, histopathology of polyp, fungal cultures, IgE level pre- and post-operative, and Phadiatop were all collected and recorded. The important variable was pre- and postoperative eosinophils count, which were studied and investigated. The percentage reference ranges of blood eosinophils in the laboratory of King Abdulaziz University Hospital, Jeddah, Saudi Arabia is 0-4%. During the period of the current investigation, steroids were not given to the patients either pre- or post-operatively.

**Figure 1:**
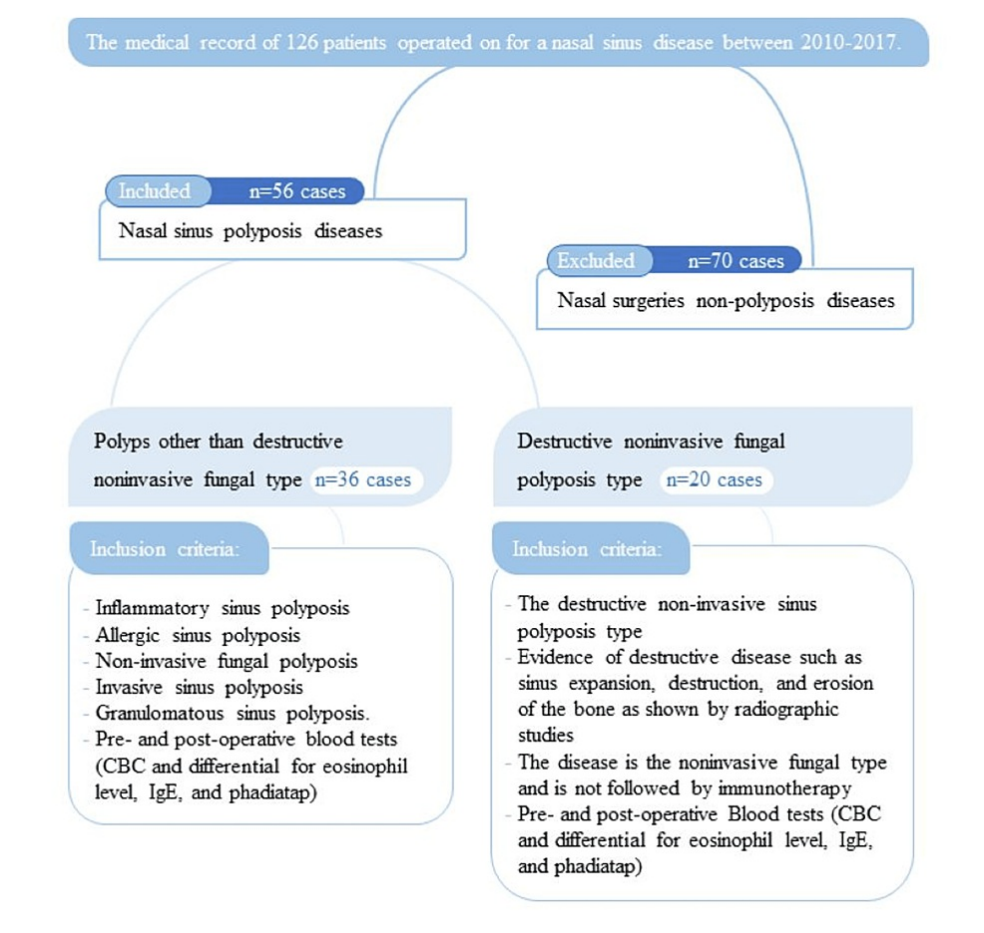
Flow chart indicating the selection process of cases. CBC: complete blood count

The selection process of cases is described in Figure 1. We selected two study groups of nasal sinus polyposis disease cases as the available data. We reviewed the role of eosinophils in the disease process, pathogenesis, and prognosis in the 20 studied cases of destructive non-invasive fungal sinus disease (Group 1) vs. 36 cases of nasal polyp diseases, including fungal and non-fungal but not destructive non-invasive fungal type (Group 2). The first group (20 cases), which included only the destructive non-invasive sinus polyposis type, was characterized by sinus expansion and bone erosion, and remodeling, rarefication, or expansion of bones were part of its destructive features. The second group (36 cases) included all types of nasal polyposis other than the destructive non-invasive sinus fungal polyposis type (inflammatory sinus polyposis, allergic sinus polyposis, non-invasive fungal polyposis, invasive sinus polyposis, and granulomatous sinus polyposis). 

Statistical methods

A nonparametric Wilcoxon signed-rank test was used to determine a significant difference at p<0.05. In addition, the statistical significance difference in pre- and post-operative eosinophil blood levels was compared in both studied groups. Since it was pre- and post-operative data on eosinophils, we also ran the multivariate analysis using repeated measure analysis of covariance (ANCOVA) and adjusted for confounders such as patient’s age, sex, pre- and post-operative IgE, fungal culture, and histopathology of the disease. Interaction between age, pre- and post-operative IgE, and proportion of eosinophils was assessed at a p-value of 0.05.

Ethical considerations

This study was approved by the Ethics Committee of King Abdulaziz University, Unit of Biomedical Ethics (ethic reference: 401-22). Informed consent was obtained from all participants prior to the commencement of the study, and the study was conducted in accordance with the Declaration of Helsinki.

## Results

The medical records of 126 patients operated on for nasal sinus disease between 2010 and 2017 were reviewed. Of the 56 cases included, 20 were of destructive non-invasive sinus fungal polyposis, which was termed Group 1, and the remaining 36 cases were other types of nasal polyposis and termed Group 2.

The demographic and disease characteristics of study participants including age, sex, histopathology, fungal cultures, and the percentage of pre- and post-operative eosinophils of 20 studied cases from Group 1 are shown in Table 1. In this retrospective study, the male-to-female ratio in Group 1 was 1:9 (90% female). The ages of the subjects ranged from 13 to 55 years, with a mean age of 28.25 years and a standard deviation of 11.68 years. In this study group, 80% of histopathology results revealed the presence of fungal organisms. Moreover, *Aspergillus* species were the most common fungi found in fungal culture, and among these cases, a significantly greater proportion (66%) was identified to be *Aspergillus flavus*.

**Table 1:** Demographic and disease characteristics of Group 1 participants -, data not available.

Serial no.	Age (years)	Sex	Histopathology	Fungal Culture	Pre-operative Eosinophils (%)	Post-operative Eosinophils (%)
1	13	Female	-	-	13.40	04.70
2	13	Female	Consistent with fungal sinusitis	*Aspergillus* species	16.30	05.70
3	20	Male	Consistent with fungal sinusitis	A. flavus	12.80	00.70
4	37	Female	Hypertrophic rhinitis+ fungal infection	A. fumigatus	06.00	04.40
5	43	Female	Allergic polyps	Negative	06.60	03.70
6	26	Female	-	-	23.60	14.40
7	47	Female	Fungal sinusitis consistent with aspergillosis	A. flavus	07.90	02.50
8	26	Female	Chronic sinusitis with fungal organisms consistent with aspergillosis	A. flavus	07.80	05.80
9	33	Female	-	A. flavus	06.20	01.10
10	33	Female	-	A. flavus	06.30	05.40
11	15	Female	-	-	09.80	03.20
12	15	Female	Fungal polyposis	-	06.80	02.00
13	23	Female	Hypertrophic poloidal changes consistent with allergic aspergillosis	A. fumigatus	07.50	00.20
14	31	Female	Inflammatory polyps	A. flavus	10.70	00.30
15	21	Male	Fungal polyp	A. nigra	04.70	04.50
16	34	Female	Allergic nasal polyp fungal organism identified	A. flavus	07.10	03.90
17	18	Female	Consistent with fungal sinusitis	Negative	04.50	00.00
18	26	Female	Inflammatory nasal polyp	A. flavus	09.60	08.80
19	55	Female	Fungal polyps consistent with aspergillosis	Negative	04.20	04.40
20	36	Female	Allergic fungal	-	03.80	00.00

Phadiatop test, pre- and post-operative IgE test, and CT scan results are shown in Table 2. Phadiatop allergy screening showed that 57.14% of the cases were negative whereas positivity was shown in 42.85% of the cases. With regard to pre- and post-operative IgE blood levels, from the limited data available (as we did not have enough data on IgE results from the Group 1 cases), we found that in six cases the pre-operative IgE blood levels were higher than post-operative. The CT scan test results revealed the presence of destructive features (characterized by sinus expansion and bone erosion) in all investigated cases, with 70% showing fungal CT scan features. We recorded a significant pre- and post-operative difference in eosinophil count in Group 1. According to the Shapiro-Wilk test of normality, the pre- and post-operative blood tests of the 20 cases in Group 1 were not distributed normally and had a p-value of <0.05. Therefore, the Wilcoxon signed rank test was done giving a p-value of 0.001. The Wilcoxon sign rank tests with a p-value of <0.05 are considered significant.

**Table 2:** Pre- and post-operative IgE level, Phadiatop, and CT scan test results of Group 1 -, data not available.

Serial no.	IgE Pre-op (KU/L)	IgE Post-op (KU/L)	Phadiatop	CT scan
1	411	190	Negative	Destructive fungal CT features
2	546	471	Negative	Destructive fungal CT features
3	2420	-	Positive	Destructive CT features
4	3092	1067	Negative	Destructive CT features
5	172	273	Negative	Destructive fungal CT features
6	103	77.1	Positive	Destructive fungal CT features
7	288	-	Negative	Destructive CT features
8	-	3408	Positive	Destructive fungal CT features
9	-	3079	Positive	Destructive fungal CT features
10	-	-	-	Destructive fungal CT features
11	-	-	Positive	Destructive fungal CT features
12	2604	2424	-	Destructive CT features
13	-	-	-	Destructive fungal CT features
14	522	-	Negative	Destructive fungal CT features
15	23	-	-	Destructive fungal CT features
16	5259	-	Positive	Destructive CT features
17	-	-	-	Destructive fungal CT features
18	349	-	-	Destructive CT features
19	-	29.8	Negative	Destructive fungal CT features
20	-	207	Negative	Destructive fungal CT features

The demographic and disease characteristics of study participants including age, sex, histopathology, fungal cultures, and the percentage of pre- and post-operative eosinophil of 36 cases of Group 2 is shown in Table 3. The male-to-female ratio in Group 2 was 1:1.12 (52.77% female). The ages of the subjects ranged from nine to 65 years, with a mean age of 39.92 years and a standard deviation of 14.99 years.

From histopathology and CT scan, we found that 39.4% of the cases were fungal type of polyps while the others were inflammatory types (Tables 3-4). Moreover, the CT scan test results revealed that 38.5% of the fungal polyposis were invasive types whereas 30.8% were granulomatous fungal polyposis. A similar proportion (30.8%) were non-destructive fungal polyposis. The fungal culture was mostly unavailable or negative apart from four cases that showed positive results. Of these cases, two were *Aspergillus* species type, and the other two were *Zygomycetes* and *Microsporum audouinii*, respectively.

**Table 3:** Demographic and disease characteristics of Group 2 participants -, data not available.

Serial no.	Age (years)	Sex	Histopathology	Fungal culture	Pre-operative eosinophils (%)	Post-operative eosinophils (%)
1	31	Female	Inflammatory polyp fungal aspergillosis noninvasive	-	05.30	20.90
2	23	Male	Allergic inflammatory polyp	Negative	10.70	01.40
3	31	Female	Allergic nasal polyp	Negative	08.40	03.30
4	28	Male	-	-	03.90	05.00
5	40	Male	Inflammatory allergic polyp	-	07.80	06.00
6	60	Male	Allergic Inflammatory polyp	-	00.00	09.10
7	59	Female	Allergic inflammatory polyp	-	02.40	16.70
8	27	Male	Allergic nasal polyp with fungal-like hyphae	-	04.50	10.00
9	28	Female	Allergic fungal	Microsporum audouinii	05.90	02.40
10	48	Female	Allergic nasal polyp	Normal flora	11.70	07.80
11	29	Female	Fungal polyp consistent with aspergillosis	Negative	05.70	00.50
12	31	Male	Allergic nasal polyp + fungal-like organisms	-	06.80	01.20
13	43	Female	-	-	00.60	00.90
14	47	Male	Inflammatory polyp	-	03.80	00.00
15	56	Male	Inflammatory polyp	Negative	00.90	03.30
16	31	Male	Inflammatory polyp	-	01.10	00.50
17	49	Female	Inflammatory polyp	-	01.40	02.30
18	30	Male	Inflammatory polyp	-	00.00	03.10
19	18	Female	Inflammatory polyp	-	01.10	01.90
20	18	Female	Allergic polyp	-	02.40	01.50
21	34	Male	Inflammatory allergic polyp	-	02.00	02.60
22	36	Female	Inflammatory polyp	-	03.40	02.40
23	60	Female	Inflammatory polyp	-	00.40	02.90
24	58	Male	Inflammatory polyp	-	02.00	02.00
25	9	Male	Inflammatory polyp	-	02.50	01.10
26	42	Female	Inflammatory polyp	Negative	02.40	01.20
27	31	Female	Inflammatory polyp and fungal stain (neg.)	Negative	03.00	00.00
28	64	Female	Allergic polyp	-	00.00	02.40
29	34	Female	Consistent with invasive aspergillosis	-	00.40	00.30
30	65	Male	Chronic sinusitis with aspergillus fungus infection	Negative	00.30	00.60
31	65	Male	-	-	00.30	00.00
32	59	Male	Fungal sinusitis consistent with mucor species mucormycosis orbital invasion	Zygomycetes	00.90	01.70
33	54	Male	Fungal granulomatous inflammatory polyp	*Aspergillus* species	00.60	00.00
34	33	Female	Granulomatous fungal with fungal spores consistent with aspergillosis	*Aspergillus* species	01.90	00.20
35	33	Female	Granulomatous fungal	-	02.10	01.30
36	33	Female	Granulomatous fungal	-	02.20	02.20

**Table 4:** Pre- and post-operative IgE test, Phadiatop test, and CT scan results for Group 2 -, data not available.

Sr. no.	IgE Pre-op (KU/L)	IgE Post-op (KU/L)	Phadiatop	CT scan
1	-	215	Positive	CT scan features of non-fungal nasal polyposis
2	-	-	-	Non-destructive fungal nasal polyposis CT features
3	-	-	-	Non-destructive fungal nasal polyposis CT features
4	-	-	-	CT scan features of non-fungal nasal polyposis
5	-	-	-	CT scan features of non-fungal nasal polyposis
6	-	624	Negative	CT scan features of non-fungal nasal polyposis
7	292	-	Negative	CT scan features of non-fungal nasal polyposis
8	-	-	-	Non-destructive fungal nasal polyposis CT features
9	1206	855	Positive	CT scan features of non-fungal nasal polyposis
10	-	86.7	Positive	CT scan features of non-fungal nasal polyposis
11	78.3	-	Negative	CT scan features of non-fungal nasal polyposis
12	-	-	-	Invasive fungal sinonasal polyposis
13	-	-	-	CT scan features of non-fungal nasal polyposis
14	-	-	-	-
15	-	-	-	-
16	-	-	-	CT scan features of non-fungal nasal polyposis
17	-	-	-	Non-fungal left sinonasal polyp
18	-	630	Positive	CT scan features of non-fungal nasal polyposis
19	-	72.9	-	CT scan features of non-fungal nasal polyposis
20	66	-	-	CT scan features of non-fungal nasal polyposis
21	161	-	Positive	CT scan features of non-fungal nasal polyposis
22	-	-		CT scan features of non-fungal nasal polyposis
23	-	165	Negative	CT scan features of non-fungal nasal polyposis
24	-	-	-	CT scan features of non-fungal nasal polyposis
25	-	-	-	CT scan features of non-fungal nasal polyposis
26	-	-	-	No CT scan
27	-	-	-	CT scan features of non-fungal nasal polyposis
28	112	91.5	Negative	Non-destructive fungal nasal polyposis CT features
29	2377	-	Positive	Invasive fungal sinonasal polyposis
30	-	-	-	Invasive fungal sinonasal polyposis
31	-	-	-	Invasive fungal sinonasal polyposis
32	-	-	-	Invasive fungal sinonasal polyposis
33	-	-	-	Fungal granulomatous polyp
34	-	-	-	Fungal granulomatous polyp
35	-	-	-	Fungal granulomatous polyp
36	-	-	-	Fungal granulomatous polyp

Phadiatop and pre- and post-operative IgE blood level results are shown in Table 4. Phadiatop allergy screening showed that 45.5% of the cases were negative while 54.5% were positive. However, for Group 2, we did not have enough data for IgE results. In general, the pre-operative IgE blood levels were higher than post-operative. We ran a normality test (Shapiro-Wilk) for data collection of pre- and post-operative eosinophils count (%) of the 36 cases in Group 2, and the normality test gave a p-value of .001 (<0.05), which means that the data was not distributed normally. Therefore, we opted for the nonparametric Wilcoxon signed-rank test. There was no significant difference between pre- and post-operative eosinophil levels (p= 0.644).

Findings of the multivariate analysis

Group 1

After running the univariate analysis, we adjusted for the confounders such as age, sex, fungal culture, pre and post IgE, and histopathology of the disease. According to the F-test statistics (Table 5), there is a significant difference in the proportion of blood eosinophils pre and post-operative after controlling for confounders such as age, sex, fungal culture, pre and post IgE, and histopathology of the disease. This implies that in Group 1, a substantial difference was found between pre-and post-operative blood eosinophil (p=0.01) even after adjusting for the confounders described above. However, as hypothesized, we did not find any significant interaction with the age of the patients, meaning that the rate of eosinophils between pre- and post-operative did not vary for patients with different ages (p-value for interaction: 0.07) or different pre- and post-operative IgE levels (p-value: 0.46).

**Table 5:** Multivariate analysis showing the difference between pre and post-operative blood eosinophils in Group 1 (n=20) df: degree of freedom The model was adjusted for confounders such as age, sex, fungal culture, pre and post IgE, and histopathology of the disease; P-value significance cut-off: 0.05 ^**^ Statistically significant p-value for difference between pre- and post-operative proportion of blood eosinophils

Variable	Type III sum of squares	df	Mean square	F-statistics	P-value
Eosinophils	75.963	1	75.963	52.737	0 .018**
Age	27.117	1	27.117	0.332	0.623
Sex	1.057	1	1.057	0.032	0.861
IgE (Preoperative)	1.518	1	1.518	0.019	0.904
IgE (Postoperative)	24.889	1	24.889	0.304	0.637
Histopathology	141.258	13	10.866	0.155	0.997
Fungal Culture	89.037	5	17.807	0.542	0.742
Eosinophils, Age	17.216	1	17.216	11.952	0.074
Eosinophils, IgE Preoperative	0.757	1	0.757	0.526	0.544
Eosinophils, IgE Postoperative	1.189	1	1.189	0.825	0.460

Group 2

According to the F-test statistics (Table 6), there is no significant difference in the proportion of blood eosinophils pre and post-operative after controlling for confounders such as age, sex, fungal culture, pre and post IgE, and histopathology of the disease. This implies that among 36 cases of Group 2, no substantial difference was found between pre-and post-operative blood eosinophils (p=0.66) even after adjusting for the confounders described above.

**Table 6:** Multivariate analysis showing the difference between pre- and post-operative blood eosinophils in Group 2 (n=36) df: degree of freedom The model was adjusted for confounders such as age, sex, fungal culture, pre and post IgE, and histopathology of the disease; P-value significance cut-off: 0.05

Variable	Type III Sum of Squares	df	Mean Square	F-Statistics	P-value
Eosinophils	6.664	1	6.664	0.256	0.663
Age	41.538	1	41.538	0.545	0.537
Sex	53.872	1	53.872	0.707	0.489
IgE (Preoperative)	1.518	1	1.518	0.019	0.904
IgE (Postoperative)	27.655	1	27.655	0.363	0.608
Histopathology	141.258	13	10.866	0.155	0.997
Fungal_Culture	86.653	2	43.327	0.569	0.637
Eosinophils, Age	8.066	1	8.066	0.310	0.634
Eosinophils, Fungal culture	92.555	2	46.278	1.777	0.360
Eosinophils, Sex	24.329	1	24.329	0.934	0.436

## Discussion

The current study evaluated the significance of eosinophils in the pathogenesis of destructive non-invasive fungal polyposis sinus disease. In several studies, eosinophils are reported to be a major player in the pathogenesis of CRS through the production of the eosinophil granule proteins, MBP, and eosinophil cationic protein (ECP), which activate mast cells during inflammation [7,14,16,18,23]. The MBP is a small, specific, cytotoxic cationic protein present in eosinophils. It has pathological effects through a direct cytotoxic allergic reaction, parasitic and fungal infections, and stimulation of inflammatory cells to produce inflammatory molecules like cytokines, chemokines, and other inflammatory mediators.

In the present study, among the patients of Group 1, *Aspergillus* species were the common fungi found in patients’ fungal culture, and also a significant presence of destructive nasal sinus CT scan features was observed. This could be explained by the fact that activated eosinophils release ECP granules stored within them, which are known to have a biological effect on host cells and microbes at the same time and they essentially play a defensive role against large, non-phagocytosed organisms like helminth and fungi [23]. These excessive reactions to protect human cells contribute to the pathology and the destructive effect of these cells [7,16]. According to several studies, the specific MBP of eosinophils has more harmful and destructive effects on the tissues [16-18].

Evidence suggests that significant overlap among the categories of eosinophil-related fungal rhinosinusitis. The patients especially manifest exaggerated cellular eosinophilic responses to the fungi or their extract. The response is to the commonly isolated fungus, A. flavus, in the local region rather than *Alternaria alternata* [24]. Moreover, the results of the current study showed that the pre-operative eosinophils count level was significantly higher than post-operative, which may be involved in the pathogenesis of the disease in Group 1. Clusters of eosinophils and fungal elements are both present in the mucus of CRS patients and appear to be a marker of CRS disease [25]. Ponikau proposed that certain fungi could elicit eosinophilic inflammation in the absence of a type Ⅰ hypersensitivity reaction in a patient with CRS [21]. Eosinophils are present in the mucosa of all selected groups of destructive types [13,14,26]. Increased blood eosinophil count is significantly related to more severe symptoms [4,27]. A simple blood test (blood eosinophil count) has been shown to be a good diagnostic and prognostic indicator [27,28]. Eosinophils have a major effect on the destructive pathogenesis of the disease process of fungal sinus polyposis [4,7,26,27]. Therefore, the high level of pre-operative blood eosinophils in Group 1 of the study indicates the significance of eosinophils on the disease processes and pathogenesis of destructive non-invasive fungal polyposis type. These findings were in line with a recent study conducted by Diaz-Cabrera et al., who reported noninvasive yet destructive allergic fungal rhinosinusitis in an immunocompetent patient [29].

With regard to Group 2 of the current study, inflammatory polyposis was more common than the fungal type. Moreover, there was not enough data regarding the fungal culture, IgE, and Phadiatop results of these cases. The CT scan findings showed that the invasive fungal type was more common in Group 2 than the granulomatous and non-destructive fungal polyposis. This study found that Group 2 had no significant difference in blood eosinophil levels between pre- and post-operative in comparison to Group 1. The findings of the eosinophils levels in destructive non-invasive fungal polyp types in this study were consistent with a study by Sreeparvathi et al., who reported that a significant correlation between tissue and blood eosinophils count in Indian patients with severe symptoms with eosinophilic CRS with nasal polyps [28].

According to several studies, serum eosinophil level can be used as an indicator in the presence of CRS and as an indicator for the line of management and prognosis [27,28]. In our study, serum eosinophil level showed a valuable indicator marker in destructive non-invasive chronic fungal rhinosinusitis type. Steroids following endoscopic sinus surgery are recommended to control eosinophilic inflammation. Steroid nasal wash must be started early post-operatively to avoid extensive and aggressive diseases [7,30].

However, our findings need to be interpreted in light of some limitations inherent to the study. First, though the medical records of 126 patients operated on for a nasal sinus disease between 2010-2017 were studied, our study found that limited data were available with regard to the IgE blood level results and the fungal culture of some patients’ cases. Second, our sample size was not large enough, and the use of a larger sample size may have allowed useful insights into the importance of eosinophils in the progression and prognosis of destructive non-invasive fungal rhinosinusitis.

## Conclusions

Eosinophils play a major role in the destructive non-invasive pathogenesis of the disease process of fungal sinus polyposis as we see from the high level of pre-operative blood eosinophils compared to post-operative level in the first study group. A positive fungal culture is considered a normal finding in nasal secretions, but clusters of eosinophils with fungal elements in the mucus of CRS patients appear to be a marker of the disease. There is a statistically significant difference in blood eosinophils count between pre- and post-operative blood levels for the findings of the first group of destructive non-invasive fungal polyposis whereas this difference is not seen in the second group, even after adjusting for potential confounders such as patient’s age, sex, pre- and post-operative IgE, fungal culture, and histopathology of the disease. The current study showed that eosinophils have a major role in the pathogenesis and the disease processes in destructive non-invasive fungal sinus polyposis. Serum eosinophilia can be considered an indicator of the disease prognosis and destructive behavior.
